# Supplementary material for: Effectiveness of Online Programmes for Family Carers of People with Intellectual Disabilities: Systematic Review of the International Evidence Base
Source: Healthcare (Basel). 2024 Jul 6;12(13):1349. doi: 10.3390/healthcare12131349 (PMC11241307; doi:10.3390/healthcare12131349)
Supplement: Supplementary file 1 [file healthcare-12-01349-s001.zip › healthcare-3042970-File S1. Medline (Ovid).pdf]

**Medline (Ovid) search strategy**<br><br>Ovid MEDLINE(R) ALL <1946 to January 18th 2024>	
(Randomized Controlled Trial or Controlled Clinical Trial or Pragmatic Clinical Trial or Clinical Study or Adaptive Clinical Trial or Equivalence Trial).pt.707293	707293
(Clinical Trial or Clinical Trial, Phase I or Clinical Trial, Phase II or Clinical Trial, Phase III or Clinical Trial, Phase IV or Clinical Trial Protocol).pt.	616221
Multicenter Study.pt.	343397
Clinical Studies as Topic/	816
exp Clinical Trial/ or exp Clinical Trials as Topic/ or Clinical Trial Protocol/ or Clinical Trial Protocols as Topic/ or exp "Clinical Trial (topic)"/	1298812
Multicenter Study/ or Multicenter Studies as Topic/ or "Multicenter Study (topic)"/	363804
Randomization/	107076
Random Allocation/	107076
Double-Blind Method/	177622
Double Blind Procedure/	0
Double-Blind Studies/	177622
Single-Blind Method/	33262
Single Blind Procedure/	0
Single-Blind Studies/	33262
Placebos/	35934
Placebo/	0
Control Groups/	2093
Control Group/	2093
Cross-Over Studies/ or Crossover Procedure/	56213
(random* or sham or placebo*).ti,ab,hw,kf.	1882166
((singl* or doubl*) adj (blind* or dumm* or mask*)).ti,ab,hw,kf.	273461
((tripl* or trebl*) adj (blind* or dumm* or mask*)).ti,ab,hw,kf.	1772

(control* adj3 (study or studies or trial* or group*)).ti,ab,hw,kf.	1984557
(clinical adj3 (study or studies or trial*)).ti,ab,hw,kf.	1473147
(Nonrandom* or non random* or non-random* or quasi-random* or quasirandom*).ti,ab,hw,kf.	57113
(phase adj3 (study or studies or trial*)).ti,ab,hw,kf.	184304
((crossover or cross-over) adj3 (study or studies or trial*)).ti,ab,hw,kf.	78459
((multicent* or multi-cent*) adj3 (study or studies or trial*)).ti,ab,hw,kf.	420785
allocated.ti,ab,hw.	87669
((open label or open-label) adj5 (study or studies or trial*)).ti,ab,hw,kf.	47045
((equivalence or superiority or non-inferiority or noninferiority) adj3 (study or studies or trial*)).ti,ab,hw,kf.	13042
(pragmatic study or pragmatic studies).ti,ab,hw,kf.	639
((pragmatic or practical) adj3 trial*).ti,ab,hw,kf.	8204
((quasiexperimental or quasi-experimental) adj3 (study or studies or trial*)).ti,ab,hw,kf.	13320
trial.ti,kf.	326061
or/1-35	3929877
exp animals/	26992805
exp animal experimentation/	10425
exp models animal/	647136
exp animal experiment/	10425
nonhuman/	0
exp vertebrate/	26234329
or/37-42	26994783
exp humans/	21794589
exp human experiment/	0
or/44-45	21794589

43 not 46	5200194
36 not 47	3522488
(Online or Internet or E-learning or Web* or Virtual or Computer-based or Electronic or Tech* or Telemedicine).mp. [mp=title, book title, abstract, original title, name of substance word, subject heading word, floating sub-heading word, keyword heading word, organism supplementary concept word, protocol supplementary concept word, rare disease supplementary concept word, unique identifier, synonyms, population supplementary concept word, anatomy supplementary concept word]	4914639
(Care* or Parent* or Famil*).mp. [mp=title, book title, abstract, original title, name of substance word, subject heading word, floating sub-heading word, keyword heading word, organism supplementary concept word, protocol supplementary concept word, rare disease supplementary concept word, unique identifier, synonyms, population supplementary concept word, anatomy supplementary concept word]	4629190
(Program* or Intervention or Training or Education*).mp. [mp=title, book title, abstract, original title, name of substance word, subject heading word, floating sub-heading word, keyword heading word, organism supplementary concept word, protocol supplementary concept word, rare disease supplementary concept word, unique identifier, synonyms, population supplementary concept word, anatomy supplementary concept word]	3161825
(Intellectual disab* or Learning disab).mp. [mp=title, book title, abstract, original title, name of substance word, subject heading word, floating sub-heading word, keyword heading word, organism supplementary concept word, protocol supplementary concept word, rare disease supplementary concept word, unique identifier, synonyms, population supplementary concept word, anatomy supplementary concept word]	74651
(Intellectual Disability or Learning Disabilities).mp. [mp=title, book title, abstract, original title, name of substance word, subject heading word, floating sub-heading word, keyword heading word, organism supplementary concept word, protocol supplementary concept word, rare disease supplementary concept word, unique identifier, synonyms, population supplementary concept word, anatomy supplementary concept word]	88872
52 or 53	90671
49 and 50 and 51 and 54	814
1 or 2 or 3 or 4 or 5 or 6 or 7 or 8 or 9 or 10 or 11 or 12 or 13 or 14 or 15 or 16 or 17 or 18 or 19 or 20 or 21 or 22 or 23 or 24 or 25 or 26 or 27 or 28 or 29 or 30 or 31 or 32 or 33 or 34 or 35 or 36 or 37 or 38 or 39 or 40 or 41 or 42 or 43 or 44 or 45 or 46 or 47 or 48	27552208
49 and 50 and 51 and 52 and 53 and 54 and 55 and 56	504
55 and 56	693

48 and 55	168
("adaptive clinical trial" or "clinical trial" or "clinical trial, phase i" or "clinical trial, phase ii" or "clinical trial, phase iii" or "clinical trial, phase iv" or "controlled clinical trial" or "equivalence trial" or "multicenter study" or "pragmatic clinical trial" or "randomized controlled trial").pt. or double-blind method/ or "adaptive clinical trials as topic"/ or "clinical trials as topic"/ or "clinical trials, phase i as topic"/ or "clinical trials, phase ii as topic"/ or "clinical trials, phase iii as topic"/ or "clinical trials, phase iv as topic"/ or "controlled clinical trials as topic"/ or "equivalence trials as topic"/ or "intention to treat analysis"/ or "non-randomized controlled trials as topic"/ or "pragmatic clinical trials as topic"/ or "randomized controlled trials as topic"/ or "multicenter studies as topic"/ or (phase adj1 ("I" or "II" or "III" or "IV" or "1" or "2" or "3" or "4")).ti,ab,kf. or ((randomi?ed adj7 trial*) or (controlled adj3 trial*) or ((clinical or pragmatic) adj2 trial*) or (research adj (studies or study)) or ((single or doubl* or tripl* or treb*) adj4 (blind* or mask*))).ti,ab,kf. or (("4" or four) adj arm).ti,ab,kf.	2087369
55 and 60	136
evaluation studies/ or "evaluation studies as topic".pt. or program evaluation/ or validation studies/ or "validation studies as topic".pt. or (effectiveness or intervention or (pre- adj5 post-) or (pretest adj5 posttest) or (program* adj6 (evaluate or evaluated or evaluates or evaluating or evaluation or evaluations or evaluator or evaluators)) or (quasi adj1 experimental)).ti,ab,kf.	1889420
60 or 62	3574530
57 and 63	238
